# Serological Evaluation of Anti- FSH antibody, and Anti- LH antibody In Iraqi women with polycystic ovarian syndrome

**DOI:** 10.12688/f1000research.172281.1

**Published:** 2025-12-03

**Authors:** Sura F. Alsaffar, Hiba M. Khaleel

**Affiliations:** 1 Biology, University of Baghdad Al-Jaderyia Campus College of Science, Baghdad, Baghdad Governorate, Iraq; 2 Biology, University of Baghdad Al-Jaderyia Campus College of Science, Baghdad, Baghdad Governorate, Iraq

**Keywords:** Polycystic Ovary Syndrome; Anti-FSH antibody; Anti-LH antibody; LH/FSH ratio.

## Abstract

**Background:**

Polycystic ovarian syndrome (PCOS) is a hormonal, metabolic disorder in women of reproductive age. It can be induced by genetic, immunological, and environmental determinants. The main pathophysiology of PCOS, is hyperandrogenism, which can lead to acne, hirsutism, menstrual irregularities, as well as infertility. However, PCOS is also associated with chronic low-grade inflammation.

**Objectives:**

This study aimed to evaluate the role of, (anti-FSH, and anti-LH antibodies, in PCOS occurrence).

**Methods:**

About 65 patients with PCOS, and 56 healthy women (controls) were recruited. Their ages were (20-45) years. Body mass index BMI was computed as, (weight) divided by (height squared, (kg/m ^2^). Blood samples were obtained randomly from the Medical City Hospital in Baghdad and private infertility clinics. This study was conducted between November 2024 and January 2025. The levels of anti-FSH and anti-LH antibodies were measured by ELISA.

**Results:**

The results revealed a non-significant increase in anti-FSH antibody in patients (36.22±6.73) ng/ml, compared to controls (34.99±9.78 ng/ml), and a highly significant decrease in anti-LH antibody in patients (45.29±3.04 ng/ml), as compared to controls (69.16±10.90 ng/ml), (p<0.05). The fertility hormone, FSH showed a non-significant increase in non- healthy women (8.12±0.74 mIU/ml), in contrast to healthy women (7.35±0.62 mIU/ml) (p>0.05). While the LH hormone results was, in patients with PCOS (7.61±0.73 mIU/ml), as opposed to the control group it was (5.62±0.56 mIU/ml), the (p>0.05). Finally, the LH/FSH ratio showed a non- significant increase in both patients (1.161±0.37) and controls (0.7819±0.22) (p>0.05).

**Conclusion:**

Women with PCOS showed increased LH levels and the LH/FSH ratio. However, a decrease in anti-LH antibody was accompanied by an increase of LH hormone and LH/FSH ratio.

## 1. Introduction

Polycystic ovarian syndrome (PCOS) is a prevalent gynecological disorder that affects women of reproductive age (15-45 years) and is characterized by a combination of hormonal and metabolic manifestations. ^1, 2^ The exact cause of this syndrome remains unclear; however, PCOS is primarily associated with hyperandrogenism and ovulatory dysfunction. Its incidence varies by region and is influenced by lifestyle factors, including diet and physical activity habits. The pathophysiology of PCOS, resulting from hyperandrogenism and insulin resistance, can lead to hirsutism, acne, and menstrual cycle irregularities, anovulation, endometrial cancer, ovarian enlargement, infertility, type 2 diabetes, other cardiovascular diseases. ^3, 4^ Environmental and genetic factors contribute to PCOS development in ovaries. Lifestyle exacerbates these conditions; therefore, lifestyle modification through a healthy diet and regular physical activity is considered the first-line approach for all PCOS phenotypes. However, its effect on ovulation and fertility in lean PCOS patients appear limited. Metabolic disorders, including insulin resistance, hypertension, dyslipidemia, and central obesity are more common in obese women with PCOS. Furthermore, scientific evidence indicates that women with PCOS have higher levels of inflammation-related biomarkers in both their serum and ovarian tissues than women without the condition. ^5, 6^ PCOS causes an imbalance in progesterone and estrogen (sex hormones) levels, which leads to disturbances in the menstrual cycle. Therefore, oral contraceptives are often prescribed for women with PCOS. ^7^ This condition can be diagnosed by ovarian ultrasonography. The ovary can be affected by immune-mediated processes, and these responses can be organ-specific, affecting only the ovary or systemic autoimmune diseases. One autoimmune conformation of the ovary, anti-FSH antibody interferes with the function of FSH, and FSH’s specific beta-epitope is predominant in endometriosis and polycystic ovary syndrome. ^8^ The pituitary gland abides by slow and fast GnRH pulses. During the early follicular stage, increasing FSH concentrations stimulate estradiol (E2) production. Elevated E2 selectively suppresses FSH secretion and sustains a high-frequency pattern of GnRH pulses into the late follicular stage. ^9^ A hallmark of PCOS is an increase in GnRH levels, leading to elevated androgens levels. The ovary stimulates this over secretion and produces high LH levels from the adenohypophysis. This elevation in LH secretion is observed in approximately 60% of women with PCOS, and over-secretion of LH prevents oocyte maturation. The main function of LH hormone, stimulate ovulation and zygote implantation in uterus, as well as to regulate the menstrual cycle. ^10, 11^ LH receptors on ovarian theca cells regulate steroid production, whereas FSH receptors on granulosa cells regulate follicle development and steroid hormone handling. FSHR and LHR share structural and evolutionary similarities with the Thyroid Stimulating Hormone Receptor (TSHR) in Grave’s disease, suggesting that they may also be targeted by autoantibodies. Evidence from limited studies indicates a possible association between these antibodies and premature ovarian failure in women. ^12^ In infertile women anti-FSH antibody were found at high concentrations. Because of their similarities in structure, function, and site of secretion, antibodies against both LH and FSH are the same. ^13^ LH, FSH, and LH/FSH ratio may serve as useful indicators for the diagnosis of PCOS. An increase in the LH/FSH ratio raising in level to 2 or 3 in women with PCOS, the typical ratio 1:1 of LH/FSH, means that the levels of both FSH and LH in the bloodstream can be compared. ^14, 15^ Pretreatment with oral contraceptives is used to balance the LH/FSH ratio before ovulation, to regulate ovarian induction. ^16^ This study aimed to determine the influence of anti-FSH, and anti-LH antibodies on PCOS occurrence.

## 2. Materials and methods

The study was conducted in Baghdad, and samples were randomly collected from the Medical City Hospital, Baghdad, and private women’s infertility clinics. Written informed consent was obtained from all patients and controls prior to participation. The study protocol was approved by the Ethics Committee of the College of Science, University of Baghdad (Ref.: CSEC/1124/0100, November 2024). A total of 65 women with PCOS and 56 healthy women were included in the study. The control group was comparable to patients in terms of age and body mass index (BMI). In addition, the range of women’s ages was between 19 and 45 years, and some patients were receiving medications such as metformin, vitamin D, and oral contraceptives as treatment protocol. Women were excluded if they were pregnant, breastfed women or had hypertension, diabetes, or other autoimmune diseases. Prior to enrollment, detailed medical histories were obtained and physical examinations were performed to confirm eligibility. The duration of treatment for patients with PCOS at the time of sample collection ranged from several months to one year. BMI was measured using the equation weight (kg)/(length)m ^2^ for both groups (patients and controls). The main symptoms that occur in patients with PCOS are (not regular period, acne, alopecia, hirsutism, oily skin, and depression). Hyperandrogenism is a key diagnostic criterion of PCOS.

### 2.1 Exclusion criteria

Pregnant, breastfeeding women, or had hypertension, diabetes, or other chronic diseases were excluded from the study.

### 2.2 Infertility hormones FSH, LH

Using the AFIAS-6 apparatus, hormones (Follicle stimulating hormone, luteinizing hormone) were measured in the serum of women with PCOS and healthy women during the early follicular phase.

### 2.3 Anti-FSH, Anti-LH antibodies

All parameters were determined using the serum from women with PCOS and healthy women (controls) during the early follicular phase. Fertility hormones (anti FSH antibody, and anti LH antibody) were measured by ELISA using immunological kits (Cat No: YLA0050HU, Shanghai YL Biotech Company, China). (Cat No: E3130Hu, BT LAB, China). The ELISA plate was precoated with human Anti-FSH antibodies. Upon adding 50 μl of the sample to the wells, any Anti-FSH Ab was bound to the immobilized antibodies. Biotin-labeled Anti-FSH Ab was dispensed into all wells except for the standard wells, which were precoated. Biotin-labeled Anti-FSH antibodies immobilized on the plate. Streptavidin-HRP was added to all wells, and the plate was kept in the dark. The mixture was incubated at 37°C for 60 min. After incubation, unbound streptavidin-HRP was washed away using washing buffer. The washing step was repeated five times, with a one- minute wait for each wash. Substrate solution (A and B) was then added, and the resulting color indicated the concentration of Anti-FSH Ab. The enzymatic reaction was stopped by adding an acidic stop solution, and absorbance was measured at 450 nm using a microplate reader. The same detection steps used for Anti-FSH Ab were applied to the Anti-LH Ab.

### 2.4 Statistical analysis

Data analysis was performed using SPSS version 2019. Statistical tests were conducted assuming approximate normality of the variables. And independent t-test was used to compare percentages. Statistical significance was set at p-value < 0.05 was considered. Simple statistical analyses were sufficient as the data did not require more complex methods.

## 3. Results

The age of patients in the PCOS group aged <30 years old was 43 (66.15%), while it was 22 (33.33%) in patients aged >30 years old. Compared to the control group less than 30 years old were 40 (71.43%), while the control group more than 30 years old were 16 (28.57%), as shown in  Table 1. The percentage of married female patients and with PCOS was 49 (75.38%), whereas the control group comprised 36 (64.29%). Comparing these results to unmarried patients 16 (24.62%), and the unmarried controls 20 (35.71%), as shown in Table 1. The patients who had children were 38 (58.46%), while those who did not have children were 27 (41.54%). As in the control group (having children) were 23 (64.29%), while control group (having no children) were 13 (35.71%), as shown in  Table 1.

**Table 1.** Distribution of sample study according to Age, in patient and control groups.

Factor		Patients No. (%)	Control No (%)	P-value
**Age groups** <30 yr.		43 (66.15%)	40 (71.43%)	
**(year)** ≥30 yr.		22 (33.85%)	16 (28.57%)	0.0395 *
	Mean ±SE	26.80 ± 0.78	27.64 ± 1.91	0.662 NS
**Married**	Married	49 (75.38%)	36 (64.29%)	0.0001 **
	Single	16 (24.62%)	20 (35.71%)	
**Children**	Have children	38 (58.46%)	23 (64.29%)	
	Don’t have children	13 (35.71%)	27 (41.54%)	0.0072 **

Means having with the different letters in same column differed significantly.

* ( *P* ≤ 0.05),

** ( *P* ≤ 0.01).

±SD = standard deviation.

There was a non-significant increase in the follicle-stimulating hormone (FSH), and luteinizing hormone (LH) in PCOS (8.12 ± 0.74 mIU/ml), (7.61 ± 0.73 mIU/ml) respectively, as compared to control (7.35 ± 0.62 mIU/ml), (5.62 ± 0.56 mIU/ml) respectively (p value > 0.05). However, LH levels showed a non-significant increase in patients when compared to controls (p > 0.05). The present study found a highly significant decrease in antibodies against LH hormone in patient (45.29 ± 3.04 ng/ml) were found to be less than control (69.16 ± 10.90 ng/ml). Serum anti-LH antibodies were detected in PCOS patients, showing a negative correlation with LH levels. The anti-FSH antibody levels showed a non-significant increase in both patient and control groups. Antibodies against FSH in patient (36.22 ± 6.73 ng/ml) was more than the control (34.99 ± 9.78 ng/ml). The LH/FSH ratio revealed a non-significant increase in the patients (1.161 ± 0.37), and control groups (0.7819 ± 0.22), as shown in  Table 2. The LH/FSH ratio was more than 2 was in patients 5(7.6%), while in control zero. The ratio was less than 2, in patient 60 (92%) higher than in control where it was 56 (100%), as shown in  Table 3.

**Table 2.** Comparison between patients and control groups in Hormones.

Means ± SE					
Groups	FSH (mIU/ml)	LH (mIU/ml)	Anti-LH Ab (ng/ml)	Anti-FSH Ab (ng/ml)	LH/FSH
Patients	8.12 ± 0.74	7.61 ± 0.73	45.29 ± 3.04	36.22 ± 6.73	1.161 ± 0.37
Control	7.35 ± 0.62	5.62 ± 0.56	69.16 ± 10.90	34.99 ± 9.78	0.7819 ± 0.22
T-test	3.297 NS	3.244 NS	16.448 **	15.530 NS	1.71
P-value	0.643	0.227	0.0050	0.936	0.09 NS

Means having the different letters in the same column differed significantly.

(mIU/ml) = milli-International Unit per milliliter.

(Ng/ml) = nanogram per milliliter.

** ( *P* ≤ 0.01), NS (Non-significant) ± SD = Standard deviation.

**Table 3.** The number and percentage of patients and control where (LH/FSH ratio >2, and LH/FSH ratio <2).

Groups	LH/FSH ratio >2	LH/FSH ratio <2
Patients	5 (7.6%)	60 (92%)
Control	0	56 (100%)

Data are presented as number and percentage (%).

The BMI of the patients and controls was as follows: in obese and overweight women the anti-LH antibody decreased in the patient (43.796) ng/ml, and control groups (69.864) ng/ml, while the anti- FSH antibody levels increased in the patients (42.561) ng/ml compared to the control group (39.653) ng/ml, and the LH/FSH ratio showed an increase in the patient (1.381), and control groups (0.825). In normal weight women, the anti-LH antibody was lower in the patients (47.586) ng/ml, than in controls (68.221) ng/ml, and the anti-FSH antibody also as anti-LH antibody, in patients (26.483) ng/ml increase, compared to control (28.791) ng/ml, while LH/FSH ratio represented a higher level in the patient (0.821) than in the control (0.723) as shown in  Table 4. There was a non-significant correlation between the BMI and (Anti-FSH, Anti-LH antibodies, and LH/FSH ratio), as shown in  Table 5.

**Table 4.** The number of patients and control BMI in anti-gonadotropin antibodies.

	BMI>25(KG/M ^2^)		BMI<25 (KG/M ^2^)	
ANTI-LH Antibody	Patient	43.796	Patient	47.586
	Control	69.864	Control	68.221
ANTI-FSH Antibody	Patient	42.561	Patient	26.483
	Control	39.653	Control	28.791
LH/FSH ratio	Patient	1.381	Patient	0.821
	Control	0.825	Control	0.723

KG/M ^2^ = kilograms per square meter.

**Table 5.** Correlation coefficient between BMI with Anti-FSH, Anti-LH and LH/FSH ratio.

Parameters	Correlation coefficient-r with BMI	
Anti-FSH	-0.09 NS	-0.04 NS
Anti-LH	0.02 NS	0.19 NS
LH/FSH ratio	0.02 NS	-0.14 NS

Means having the different letters in the same column differed significantly. NS (non-significant).

## 4. Discussion

Polycystic ovarian syndrome can be defined as a hormonal, metabolic, and endocrine disorder, related to irregular androgen production from the ovaries. An increase in adrenal androgen levels, combined with obesity, promotes the peripheral conversion to estrogen. ^17, 18^ In addition to type 2 diabetes, women with PCOS often exhibit insulin resistance, impaired glucose tolerance, and obesity. ^19^ It was normal to increase the level of LH hormone, and the ratio of LH/FSH, it may be between 1-2 in healthy women but increases to reach as high as 2-3 that will lead to anovulation. The gonadotrophin- axis was distributed in PCOS patients, so LH levels increased and FSH levels were within the normal range, leading to the expression of the normal LH/FSH ratio. This result is not concordant with that reported by Malini and Roy. ^20^ The Results of this study showed an increase in the LH/FSH ratio in female with PCOS, which increased above 1, except in some patients who were, taking an oral contraceptive that balanced the LH/FSH level before the ovulation, which may have affected the results. Despite the potential influence of oral contraceptives on the LH/FSH ratio, a small proportion (7.5%) of the patients still exhibited values above 2, which is a hallmark feature of PCOS. This finding further supports the notion that, in addition to endocrine and metabolic disturbances, immune-mediated mechanisms such as the presence of anti-LH Ab and anti-FSH Ab, may contribute to ovarian dysfunction and reproductive impairment in affected women. In addition, findings indicated an elevation in LH concentrations, along with either normal or reduced FSH concentrations, in individuals with PCOS, as revealed by previous research. ^21^ The medications that some patients with PCOS were taking are vitamin D3, oral contraceptives, and metformin when samples were collected, the role of metformin and vitamin D in PCOS was to decrease the FSH level in women during post-menopause, and decreased LH levels in women with PCOS. ^22^ Some patients had been taking medications, such as metformin and vitamin D, which were prescribed by their physician at the time of sample collection. This treatment is commonly used in PCOS to improve hormonal balance by lowering LH levels and increasing FSH levels, thereby supporting ovulation and enhancing menstrual regularity. Additionally, metformin and vitamin D have a positive effect on BMI, and their combination is particularly effective in managing ovulatory dysfunction in patients with PCOS. ^23^ Insulin resistance in women with PCOS is reduced by metformin, thereby counteracting adverse metabolic and hormonal disturbances. This improvement is accompanied by enhanced GnRH pulsatility, decreased LH secretion, and reduced androgen production, and an increase in sex hormone-binding globulin (SHBG). Normalization of LH levels and the LH/FSH ratio in this manner is considered one of the most prominent therapeutic effects of metformin on fertility in women with PCOS. ^24^ FSH and LH are inhibited by anti-FSH antibody or anti-LH antibody trapping the hormones in the immune complex, and anti-FSH antibody prevents FSH hormone from binding to its receptor. ^25^ An earlier study showed that there was a higher level of anti-FSH antibody in (patients) with PCOS than (controls), the exact mechanism remains unclear, and a previous study suggested that the epitope of the beta- chain of FSH attacked by anti-FSH antibody, these Autoantibodies made the tissue of ovaries and the hormone receptors as target, where activated the gonadotropin-releasing hormone receptor, and disrupted the action of the hypothalamic-pituitary ovarian axis. The rate of anti-FSH antibody in this study, increased in female with PCOS, which is consistent with a previous study showing an elevation in anti-FSH antibody levels in women with PCOS, However, the anti-LH antibody level did not show that increase. As well as the current study showed a non-significant relationship between (BMI) and (anti-FSH antibody, anti-LH antibody, and the LH/FSH ratio). There was a non-significant decrease in anti-LH antibody levels, in obese patients and a non-significant increase in obese controls women. The results were the same for the non-obese (patients and controls). In obese and non-obese (patients and controls), the anti-FSH antibody, LH/FSH ratio, revealed a non-significant increase, as shown in earlier research. ^26^ In earlier study, alteration in reproductive hormones were identified in PCOS patients, with FSH levels being lower and LH levels elevated. Approximately, 64.5% of the women exhibited abnormal hormonal profiles involving FSH, LH, along with an increased LH/FSH ratio. The ratio of LH/FSH showed an inverse association with body mass index. Also, these hormonal changes, particularly the imbalance in LH, FSH, and their ratio, were related to clinical manifestations of PCOS, such as obesity, insulin resistance, and hyperandrogenism. ^27^ Previous study reported elevated LH levels in PCOS patients accompanied by detectable anti-LH antibodies. These findings suggest that serum anti-LH Abs could serve as a potential marker for PCOS. In line with this, the current study observed a negative correlation between LH and anti-LH Abs, supporting the proposed relationship. ^28^ As such, this study suggested a group of patients with PCOS and controls (healthy women), according to their ages, and if they have children (fertile), or not (infertile) to detect the fertility beside that (secondary infertility), and (primary infertility) in female with PCOS was clearly increased. However, the levels of anti-LH and anti-FSH antibody in primary infertile patients with PCOS were higher than those in secondary infertile patients with PCOS and controls. To review past research, infertility was defined as a primary infertility (when couples never had children for more than 12 months). Secondary infertility (when couples never had children after the last child they had, for more than 12 months), and the female was diagnosed with PCOS without taking any treatment. BMI is a numerical value that estimates a person’s body fat based on their weight and height, BMI in obese and overweight women is above 25, whereas that in low fat women is below 25. In an earlier study the women with a BMI less than 25 (normal weight) had fewer menstrual cycle irregularities than obese and overweight women. ^29^

## 5. Conclusion

This study suggests that immune-mediated mechanisms, including increased in levels of anti-FSH antibodies, may contribute to hormonal imbalance and reproductive dysfunction in women with PCOS. An elevated LH level and LH/FSH ratio above 2 were observed in the PCOS group, with cases of primary and secondary infertility clear. Endocrine and metabolic factors remain important, considering that immune factors could improve patient assessment and fertility management. Future studies with larger sample sizes and advanced analyses are recommended to better understand these interactions and to guide targeted interventions.

## Ethical statement

This study was approved by the Ethics Committee of the College of Science, University of Baghdad (Ref.: CSEC/1124/0100, November 2024). All procedures involving human participants were conducted in accordance with the ethical standards of the Declaration of Helsinki. Written consent was obtained from each patient and control before the study started.

## Data Availability

All data supporting the findings of this study are available in the Zendo repository. The raw data of the paper was uploaded to zendo repository in the following DOI: https://doi.org/10.5281/zenodo.17499229 ^30^ 2- Zendo: questionnaire and written consent form were uploaded as mentioned in the following Doi: https://doi.org/10.5281/zenodo.17499229 ^30^ Data is available under Creative Commons Attribution 4.0 International license (CC-BY 4.0).
