# Serological Evaluation of Anti- FSH Antibody and Anti- LH antibody In Iraqi Women with Polycystic Ovarian Syndrome

**DOI:** 10.12688/f1000research.172281.5

**Published:** 2026-07-30

**Authors:** Hiba M. Khaleel, Sura F. Alsaffar

**Affiliations:** 1 Biology, University of Baghdad Al-Jaderyia Campus College of Science, Baghdad, Baghdad Governorate, Iraq; 2 Biology, University of Baghdad Al-Jaderyia Campus College of Science, Baghdad, Baghdad Governorate, Iraq

**Keywords:** Polycystic Ovary Syndrome; Anti-FSH antibody; Anti-LH antibody; LH/FSH ratio.

## Abstract

**Background:**

Polycystic ovarian syndrome (PCOS) is a hormonal, metabolic disorder in women of reproductive age. It can be induced by genetic, immunological, and environmental determinants. The main pathophysiology of PCOS, is hyperandrogenism, which can lead to acne, hirsutism, menstrual irregularities, as well as infertility. However, PCOS is also associated with chronic low-grade inflammation. There was little study on the autoimmunity, like anti-FSH and anti-LH antibodies, of PCOS, so the current study was designed to test the effectiveness of these antibodies on the occurrence of PCOS.

**Objectives:**

This study aimed to compare the serum levels of anti-gonadotropin antibodies (anti-FSH, and anti-LH antibodies) between women with PCOS and healthy controls; and to evaluate their association with BMI status.

**Methods:**

A total of 65 patients with PCOS, and 56 healthy women (controls) were recruited. Their ages were 18-45 years. Body mass index (BMI) was computed as, (weight) divided by (height squared, (kg/m ^2^). Blood samples were obtained from both patients and controls during the early follicular phase (day 2-3 of the menstrual cycle) using convenience sampling from the Medical City Hospital in Baghdad and private infertility clinics. PCOS was diagnosed according to the Rotterdam criteria (2003). This study was conducted between November 2024 and January 2025. The levels of anti-FSH and anti-LH antibodies were measured by ELISA.

**Results:**

The results revealed a non-significant increase in anti-FSH antibody in patients (36.22±6.73) ng/ml, compared to controls (34.99±9.78 ng/ml), and a highly significant decrease in anti-LH antibody in patients (45.29±3.04 ng/ml), as compared to controls (69.16±10.90 ng/ml), (p<0.05). The fertility hormone, FSH showed a non-significant increase in unhealthy women (8.12±0.74 mIU/ml), in contrast to healthy women (7.35±0.62 mIU/ml) (p>0.05). While the LH hormone results was, in patients with PCOS (7.61±0.73 mIU/ml), as opposed to the control group, which was (5.62±0.56 mIU/ml), the (p>0.05). Finally, the LH/FSH ratio showed a non-significant increase in both patients (1.161±0.37) and controls (0.7819±0.22) (p>0.05). There was a non-significant correlation between BMI and anti-LH or anti-FSH antibodies (p >0.05).

**Conclusion:**

Women with PCOS exhibit a significant decrease in anti-LH antibody levels along with variations in LH levels and LH/FSH ratio compared to healthy controls.

## 1. Introduction

Polycystic ovarian syndrome (PCOS) is a prevalent gynecological disorder that affects women of reproductive age (15-45 years) and is characterized by a combination of hormonal and metabolic manifestations. ^1, 2^ The exact cause of this syndrome remains unclear; however, PCOS is primarily associated with hyperandrogenism and ovulatory dysfunction. Its incidence varies by region and is influenced by lifestyle factors, including diet and physical activity habits. The pathophysiology of PCOS, resulting from hyperandrogenism and insulin resistance, can lead to hirsutism, acne, and menstrual cycle irregularities, anovulation, endometrial cancer, ovarian enlargement, infertility, type 2 diabetes, other cardiovascular diseases. ^3, 4^ Environmental and genetic factors contribute to PCOS development in ovaries. Metabolic disorders, including insulin resistance, hypertension, dyslipidemia, and central obesity, are more common in obese women with PCOS. Furthermore, scientific evidence indicates that women with PCOS have higher levels of inflammation-related biomarkers in both their serum and ovarian tissues than women without the condition. ^5, 6^ PCOS causes an imbalance in progesterone and estrogen (sex hormones) levels, which leads to disturbances in the menstrual cycle. Therefore, oral contraceptives are often prescribed for women with PCOS. ^7^ This condition can be diagnosed by ovarian ultrasonography. The ovary can be affected by immune-mediated processes, and these responses can be organ-specific, affecting only the ovary, or systemic autoimmune diseases. One autoimmune conformation of the ovary, anti-FSH antibody interferes with the function of FSH, and FSH’s specific beta-epitope is predominant in endometriosis and polycystic ovary syndrome. ^8^ The pituitary gland abides by slow and fast GnRH pulses. During the early follicular stage, increasing FSH concentrations stimulate estradiol (E2) production. Elevated E2 selectively suppresses FSH secretion and sustains a high-frequency pattern of GnRH pulses into the late follicular stage. ^9^ A hallmark of PCOS is an increase in GnRH levels, leading to elevated androgen levels. The ovary stimulates this oversecretion and produces high LH levels from the adenohypophysis. This elevation in LH secretion is observed in approximately 60% of women with PCOS, and oversecretion of LH prevents oocyte maturation. The main function of LH hormone is to stimulate ovulation and zygote implantation in the uterus, as well as to regulate the menstrual cycle. ^10, 11^ LH receptors on ovarian theca cells regulate steroid production, whereas FSH receptors on granulosa cells regulate follicle development and steroid hormone handling. Evidence from limited studies indicates a possible association between these antibodies and premature ovarian failure in women. ^12^ In infertile women anti-FSH antibodies were found at high concentrations. Because of their similarities in structure, function, and site of secretion, antibodies against both LH and FSH are the same. Pretreatment with oral contraceptives is used to balance the LH/FSH ratio before ovulation, to regulate ovarian induction. ^13^ There was very little previous studies on the role of anti-FSH and anti-LH antibodies in PCOS occurrence so the study was designed to compare the levels of these antibodies and its correlation with imbalanced FSH and LH levels and association with BMI in PCOS patients.

## 2. Materials and methods

### 2.1 Patients sampling

A study was carried out in Baghdad, about 65 women with PCOS and 56 healthy women were included in the study. The women’s ages ranged between 18 and 45 years in patients and controls, about four participants were on treatment for PCOS like metformin, vitamin D, and oral contraceptives during sample collection, but they continued to meet the diagnostic criteria for PCOS. The doses of medications were not standardized among participants. The samples were collected using a clinical convenience sampling technique from the Medical City Hospital, Baghdad, and private women’s infertility clinics. Written consent was obtained from each patient and control before the study started. The study was approved by the Ethics Committee of the College of Science, University of Baghdad (Ref.: CSEC/1124/0100, November 2024 to Jan 2025). Due to the strict eligibility criteria and logistic challenges in recruiting completely healthy individuals, the sample size of the control group was smaller than the PCOS group. However, to minimize potential confounding factors, controls were closely matched with patients based on age. BMI was measured using the equation weight (kg)/(length).m ^2^. The cut off value was 25 as BMI < 25 is normal weight, BMI 25-30 is overweight and BMI >30 act as obese. The main symptoms that occur in patients with PCOS are irregular periods, acne, alopecia, hirsutism, oily skin, and depression. Hyperandrogenism is a key diagnostic criterion of PCOS. To verify the adequacy of the final sample size (65 cases and 56 controls), a post-hoc power analysis was conducted using G*Power software. Based on current sample size, the study achieved a statistical power (1-β) of over 80% (>0.80) at a significant level (α) of 0.05, confirming that the sample size is statistically sufficient to detect significant differences between the groups.

The sample size was calculated by Raosoft; the margin of error is 5%, the confidence level was 95% and response of distribution was 50%.

### 2.2 Diagnostic criteria

The diagnosis of PCOS was established based on the Revised Rotterdam Consensus criteria, requiring the presence of at least two of the following components: Oligo- or anovulation, clinical and/or biochemical hyperandrogenism, and PCOS morphology on ultrasound. In this study, the diagnostic criteria are classified as follows: Clinical criteria (based on patients’ symptoms, physical examination, and medical history), laboratory criteria (based on blood tests, hormonal levels, and immunological assays, including antibody detection), and imaging criteria (based on radiological and imaging techniques such as ultrasound). Specifically, the operational definition included assessing irregular periods or chronic amenorrhea for ovulatory dysfunction, and evaluating hirsutism, acne, or alopecia alongside serum androgen levels for hyperandrogenism.

Anti-mullerian hormone (AMH) was assessed as part of the laboratory diagnostic criteria to support the evaluation of ovarian function. Additionally, while the LH/FSH ratio is not officially recognized as a primary diagnostic criterion within the Rotterdam guidelines, LH and FSH may serve as useful indicator for PCOS. An increase in the LH/FSH ratio to 2 or 3 in women with PCOS, the typical ratio being 1:1 of LH/FSH, means that the levels of both FSH and LH in the bloodstream can be compared. ^14, 15^ Therefore, both AMH levels and the LH/FSH ratio were utilized as supplementary pathophysiological markers rather than primary diagnostic tools. The phenotypes were not specific in this study, as the analysis focused on overall diagnosis rather than phenotype classification.

### 2.3 Exclusion criteria

Pregnant or breastfeeding women or those who had hypertension, diabetes, or other chronic diseases were excluded from the study. Also, individuals with thyroid dysfunction, hyperprolactinemia, and other endocrine conditions that could influence hormonal or immunological parameters were excluded from the study to minimize confounding factors. Only confirmed cases of PCOS were included in the study.

### 2.4 Gonadotropins hormones FSH, LH

Using the AFIAS-6 apparatus, hormones (Follicle stimulating hormone, luteinizing hormone) were measured in the serum of women with PCOS and healthy women during the early follicular phase.

### 2.5 Anti-FSH, Anti-LH antibodies

All parameters were determined using the serum from women with PCOS and healthy women (controls) during the early follicular phase. Fertility hormones (anti-FSH antibody and anti-LH antibody) were measured by ELISA using immunological kits (Cat No: YLA0050HU, Shanghai YL Biotech Company, China) (Cat No: E3130Hu, BT LAB, China). The ELISA plate was precoated with human anti-FSH antibodies. Upon adding 50 μl of the sample to the wells, any anti-FSH Ab was bound to the immobilized antibodies. Biotin-labeled anti-FSH Ab was dispensed into all wells except for the standard wells, which were precoated. Biotin-labeled anti-FSH antibodies immobilized on the plate. Streptavidin-HRP was added to all wells, and the plate was kept in the dark. The mixture was incubated at 37°C for 60 min. After incubation, unbound streptavidin-HRP was washed away using washing buffer. The washing step was repeated five times, with a one-minute wait for each wash. Substrate solution (A and B) was then added, and the resulting color indicated the concentration of anti-FSH Ab. The enzymatic reaction was stopped by adding an acidic stop solution, and absorbance was measured at 450 nm using a microplate reader. The same detection steps used for anti-FSH Ab were applied to the anti-LH Ab. According to manufacturers' specifications, the intra-assay and inter-assay coefficients of variation (CV) for both anti-LH and anti-FSH ELISA kits were <8% and <10%, respectively, confirming the precision and reproducibility of the immunological assays.

### 2.6 Statistical analysis

Data analysis was performed using SPSS version 2019. The normality of continuous variables was formally evaluated using the Shapiro-Wilk test prior to statistical analyses. An independent t-test was used to compare means between patients and controls, statistical significance was set at p-value < 0.05. While the chi-square test was used to compare percentages between groups. Simple statistical analyses were sufficient as the data did not require more complex methods. The chi-square test was used to compare the categorical variables/proportions between groups in this study. A post hoc power analysis was performed using the observed differences in Anti-LH antibody levels between PCOS patients and controls. With 65 patients and 56 controls, the statistical power was >99% at a significance level of 0.05, indicating that the sample size was adequate to detect significant differences between groups.

## 3. Results

The baseline demographic characteristics of the studied population are summarized in Table 1. The mean age of PCOS patients was 26.80 ± 0.78 years, which was statistically comparable to that of the healthy control group (27.64 ± 1.91 years; p = 0.660), demonstrating successful age-matching. Regarding marital status, a significantly higher percentage of PCOS patients were married (75.38%, n = 49) compared to the controls (64.29%, n = 36), whereas single participants constituted (24.62%, n = 16) of patients (35.71% n = 13) and of controls (p = 0.0001).

**Table 1.** Baseline demographic data (Mean ± SE) and statistical comparisons of the studied population.

Factor		Patients 65 (%)	Control 56 (%)	P-value
**Age ranges (year)**	<30	43 (66.15%)	40 (71.43%)	
	≥30	22 (33.85%)	16 (28.57%)	
	Mean ± SE	26.80 ± 0.78	27.64 ± 1.91	0.660
**Marital status**	Married	49 (75.38%)	36 (64.29%)	0.0001
	Single	16 (24.62%)	20 (35.71%)	
**Have children**	Yes	29 (58.46%)	23 (64.29%)	0.830
	No	20 (41.54%)	13 (35.71%)	

Note: Data are represented as mean ± SE for continuous variables, and as counts and percentage (%) for categorical variables. Statistical significance was set at p < 0.05.

Regarding fertility distribution, (58.46%, n = 29) of patients had children, while (41.54%, n = 20) did not. In the control group (64.29%, n = 23) had children and (35.71%, n = 13) did not. Chi-square analysis revealed that this slight difference in the prevalence of having children between the two groups was not statistically significant (p = 0.830), indicating a homogenous distribution of baseline parity.

There was a non-significant increase in the follicle-stimulating hormone (FSH), and luteinizing hormone (LH) in PCOS (8.12 ± 0.74 mIU/ml), (7.61 ± 0.73 mIU/ml) respectively, as compared to control (7.35 ± 0.62 mIU/ml), (5.62 ± 0.56 mIU/ml) respectively (p value > 0.05). However, LH levels showed a non-significant increase in patients when compared to controls (p > 0.05). The present study found a highly significant decrease in antibodies against LH hormone in patient (45.29 ± 3.04 ng/ml) were found to be less than control (69.16 ± 10.90 ng/ml). Serum anti-LH antibodies were detected in PCOS patients, showing a negative correlation with LH levels. The anti-FSH antibody levels showed a non-significant increase in both patient and control groups. Antibodies against FSH in patient (36.22 ± 6.73 ng/ml) was more than the control (34.99 ± 9.78 ng/ml). The LH/FSH ratio revealed a non-significant increase in the patients (1.161 ± 0.37), and control groups (0.780 ± 0.22), as shown in  Table 2. The LH/FSH ratio was more than 2 was in patients 5(7.6%), while in control zero. The ratio was less than 2, in patient 60 (92%) higher than in control where it was 56 (100%).

**Table 2.** Comparison of Serum Gonadotropin Levels (FSH, LH), Anti-Gonadotropin antibodies (anti-FSH, anti-LH) and LH/FSH Ratio Between Patients and Control Groups (Mean ± SE).

Group	FSH (mUI/ml)	LH (mUI/mL)	LH/FSH	Anti-FSH Ab (ng/ml)	Anti-LH Ab (ng/ml)
Patients (n = 5) Mean ± SE	8.12 ± 0.74	7.61 ± 0.73	1.161 ± 037	36.22 ± 6.73	45.29 ± 3.04
Control (n = 6) Mean ± SE	7.35 ± 0.62	5.62 ± 0.56	0.780 ± 0.2	34.99 ± 9.78	69.16 ± 10.90
t-test	3.30	3.20	1.70	15.50	16.40
P-value	0.643 (NS)	0.227 (NS)	0.09 (NS)	0.936 (NS)	0.005

Note: Data are presented as mean ± standard error (SE).

t: Independent sample t-test statistic; p: Significance value. NS = Non-significant (p > 0.05).

The statistical sugnificance level was set at (p > 0.05)

mIU/ml = milli-International Unit per milliliter.

Ng = nanogram per milliliter.

The BMI of the patients and controls was as follows: in obese and overweight women the anti-LH antibody decreased in the patient (43.80)%, and control groups (69.90)%, while the anti- FSH antibody levels increased in the patients (42.60)% compared to the control group (39.70)%, and the LH/FSH ratio showed an increase in the patient (1.381), and control groups (0.80)%. In normal weight women, the anti-LH antibody was lower in the patients (47.586) ng/ml, than in controls (68.22)%, and the anti-FSH antibody also as anti-LH antibody, in patients (26.48)% increase, compared to control (28.80)%, while LH/FSH ratio represented a higher level in the patient (0.80)% than in the control (0.80)% as shown in Table 3. There was a non-significant correlation between the BMI and (Anti-FSH, Anti-LH antibodies, and LH/FSH ratio), as shown in  Table 4.

**Table 3.** Classification of the Prevalence of Anti-Gonadotropin Antibodies and LH/FSH Ratio according to BMI in the Studied Group.

Parameters	Group	BMI > 25 (kg/m ^2^) (Overweight/Obese)	BMI<25(kg/m ^2^) Normal weight
ANTI-LH antibody	Case	43.80%	47.60%
	Control	69.90%	68.22%
ANTI-FSH antibody	Case	42.60%	26.48%
	Control	39.70%	28.80%
LH/FSH ratio	Case	1.38%	0.80%
	Control	0.90%	0.80%

Note: Data are presented as prevalence percentages (%) calculated independently within each stratified body mass index (BMI) subgroup relative to the total number of subjects in each respective group (case and controls). Due to the descriptive nature of this exploratory stratification and the small sample size within these specific individual subgroups, these values represent baseline clinical trends.

**Table 4.** Correlation coefficient (pearson r) between BMI and anti-FSH, anti-LH and LH/FSH ratio.

Parameters	Patients r (n = 65)	Controls r (n = 56)	Significance (p-value)
Anti-FSH	-0.09	-0.04	NS
Anti-LH	0.02	0.19	NS
LH/FSH ratio	0.02	-0.14	NS

Note: r: Pearson correlation coefficient. NS = non-significant (p > 0.05). The statistical significance level for all correlation analyses was maintained at p < 0.05.

## 4. Discussion

Polycystic ovarian syndrome can be defined as a hormonal, metabolic, and endocrine disorder, related to irregular androgen production from the ovaries. An increase in adrenal androgen levels, combined with obesity, promotes the peripheral conversion to estrogen. ^16, 17^ In addition to type 2 diabetes, women with PCOS often exhibit insulin resistance, impaired glucose tolerance, and obesity. ^18^ It was normal to increase the level of LH hormone, and the ratio of LH/FSH; it may be between 1-2 in healthy women but increases to reach as high as 2 or 3, which will lead to anovulation. The gonadotrophin- axis was distributed in PCOS patients, so LH levels increased and FSH levels were within the normal range, leading to the expression of the normal LH/FSH ratio. This result is not concordant with that reported by Malini and Roy. ^19^ The results of this study showed an increase in the LH/FSH ratio in female with PCOS, which increased above 1, except in some patients who were, taking an oral contraceptive that balanced the LH/FSH level before the ovulation, which may have affected the results. Despite the potential influence of oral contraceptives on the LH/FSH ratio, a small proportion (7.5%) of the patients still exhibited values above 2, which is a hallmark feature of PCOS. This finding further supports the notion that, in addition to endocrine and metabolic disturbances, immune-mediated mechanisms, such as the presence of anti-LH Ab and anti-FSH Ab, may contribute to ovarian dysfunction and reproductive impairment in affected women. In addition, findings indicated an elevation in LH concentrations, along with either normal or reduced FSH concentrations, in individuals with PCOS, as revealed by previous research. ^20^ The medications that some patients with PCOS were taking were vitamin D3, oral contraceptives, and metformin when samples were collected, but they continued to meet the diagnostic criteria for PCOS. The role of metformin and vitamin D in PCOS was to decrease the FSH level in women during post-menopause and decrease LH levels in women with PCOS. ^21^ Some patients (approximately 4 participants) had been taking medications, such as metformin and vitamin D, which were prescribed by their physician at the time of sample collection. This treatment is commonly used in PCOS to improve hormonal balance by lowering LH levels and increasing FSH levels, thereby supporting ovulation and enhancing menstrual regularity. Additionally, metformin and vitamin D have a positive effect on BMI, and their combination is particularly effective in managing ovulatory dysfunction in patients with PCOS. ^22^ Pretreatment with oral contraceptives is used to balance the LH/FSH ratio before ovulation to regulate ovarian induction. ^23^ Insulin resistance in women with PCOS is reduced by metformin, thereby counteracting adverse metabolic and hormonal disturbances. This improvement is accompanied by enhanced GnRH pulsatility, decreased LH secretion, and reduced androgen production, and an increase in sex hormone-binding globulin (SHBG). Normalization of LH levels and the LH/FSH ratio in this manner is considered one of the most prominent therapeutic effects of metformin on fertility in women with PCOS. ^24^ FSH and LH are inhibited by anti-FSH antibody or anti-LH antibody trapping the hormones in the immune complex, and anti-FSH antibody prevents FSH hormone from binding to its receptor. ^25^ An earlier study showed that there was a higher level of anti-FSH antibody in patients with PCOS than in controls. The exact mechanism remains unclear, and a previous study suggested that the epitope of the beta-chain of FSH attacked by anti-FSH antibodies. These autoantibodies made the tissue of ovaries and the hormone receptors as target, which activated the gonadotropin-releasing hormone receptor, and disrupted the action of the hypothalamic-pituitary-ovarian axis. The rate of anti-FSH antibody in this study, increased in female with PCOS, which is consistent with a previous study showing an elevation in anti-FSH antibody levels in women with PCOS. However, the anti-LH antibody level did not show that increase. As well as the current study showed a non-significant relationship between (BMI) and (anti-FSH antibody, anti-LH antibody, and the LH/FSH ratio). There was a non-significant decrease in anti-LH antibody levels, in obese patients and a non-significant increase in obese control women. The results were the same for the non-obese (patients and controls). In obese and non-obese (patients and controls), the anti-FSH antibody, LH/FSH ratio, revealed a non-significant increase, as shown in earlier research. ^26^ In earlier study, alterations in reproductive hormones were identified in PCOS patients, with FSH levels being lower and LH levels elevated. Approximately, 64.5% of the women exhibited abnormal hormonal profiles involving FSH, LH, along with an increased LH/FSH ratio. The ratio of LH/FSH showed an inverse association with body mass index. Also, these hormonal changes, particularly the imbalance in LH, FSH, and their ratio, were related to clinical manifestations of PCOS, such as obesity, insulin resistance, and hyperandrogenism. ^27^ A previous study reported elevated LH levels in PCOS patients accompanied by detectable anti-LH antibodies. These findings suggest that serum anti-LH Abs could serve as a potential marker for PCOS. In line with this, the current study observed an inverse relationship between LH and anti-LH Abs, as the results showed elevated LH levels accompanied by decreased levels of anti-LH antibodies supporting the proposed relationship. ^28^ As such, this study suggested a group of patients with PCOS and controls (healthy women), according to their ages, and if they have children (fertile), or not (infertile), to detect the fertility beside that (secondary infertility), and (primary infertility) in women with PCOS was clearly increased. However, the levels of anti-LH and anti-FSH antibody in primary infertile patients with PCOS were higher than those in secondary infertile patients with PCOS and controls. To review past research, infertility was defined as primary infertility (when couples never had children for more than 12 months). Secondary infertility (when couples never had children after the last child they had, for more than 12 months), and the female was diagnosed with PCOS without taking any treatment. BMI is a numerical value that estimates a person’s body fat based on their weight and height; BMI in obese and overweight women is above 25, whereas that in low fat women is below 25. In an earlier study the women with a BMI less than 25 (normal weight) had fewer menstrual cycle irregularities than obese and overweight women. ^29^

Non-significant differences appeared among anti-LH antibodies, anti-FSH antibodies, and the LH/FSH ratio with BMI in this study.

Some patients (approximately 4 participants) were on medications (e.g., metformin and contraceptives) during sample collection; nevertheless, they continued to meet the diagnostic criteria for PCOS as confirmed by a specialist. Based on the present findings, several recommendations for future research can be proposed: assess additional immunological markers and apply a more detailed BMI classification.

## 5. Conclusion

This study suggests that immune-mediated mechanisms, including alterations in anti-FSH antibody profiles, may play a role alongside endocrine and metabolic factors in the pathophysiology of PCOS. Although an elevated LH/FSH ratio > 2 was clinically observed in a small subset of patients, the distribution of fertility status and baseline parity did not demonstrate statistically significant variations between the studied groups. Incorporating immunological markers alongside standard hormonal screening could improve descriptive patients’ assessment. Future studies with larger sample sizes and broader longitudinal cohorts are required to establish clear causal pathways and guide targeted interventions.

## Ethical statement

This study was approved by the Ethics Committee of the College of Science, University of Baghdad (Ref.: CSEC/1124/0100, November 2024). All procedures involving human participants were conducted in accordance with the ethical standards of the Declaration of Helsinki. Written consent was obtained from each patient and control before the study started.

## Data Availability

All data supporting the findings of this study are available in the Zendo repository. 1. The raw data of the paper was uploaded to zendo repository in the following DOI: https://doi.org/10.5281/zenodo.17499229 ^30^ 2. Zendo: questionnaire and written consent form were uploaded as mentioned in the following DOI: https://doi.org/10.5281/zenodo.17499229 ^30^ Data is available under the Creative Commons Attribution 4.0 International license (CC-BY 4.0).
