# Supplementary material for: Serological Evaluation of Anti- FSH Antibody and Anti- LH antibody In Iraqi Women with Polycystic Ovarian Syndrome
Source: F1000Res. 2026 Jul 30;14:1355. Originally published 2025 Dec 3. [Version 5] doi: 10.12688/f1000research.172281.5 (PMC13638218; doi:10.12688/f1000research.172281.5)
Supplement: Peer review history

# Reviewer report: Reviewer response for version 5

**DOI:** 10.5256/f1000research.206706.r514936

**Published:** 2026-10-01

**Authors:** Amal Umran Mosa

**Affiliations:** 1 University of Kerbala, Kerbala, Iraq

The manuscript addresses an interesting aspect of PCOS . a few specific issues should be corrected:

 1. Table 2 should be rechecked, because it reports Patients (n = 5) and Control (n = 6)  although the study includes 65 patients and 56 controls. The table also states significance as (p > 0.05,) while the Methods section uses p < 0.05.

 2. The reported negative correlation between LH and anti-LH antibodies should be supported statistically  because the corresponding correlation coefficient and p-value are not presented.

 3. Medication use should be acknowledged as a limitation  a small number of participants were receiving metformin, vitamin D  or oral contraceptives during sample collection.

 4. The conclusion should be slightly moderated because anti-FSH differences were not statistically significant whereas anti-LH showed the clearer significant finding.

Is the work clearly and accurately presented and does it cite the current literature?

Yes

If applicable, is the statistical analysis and its interpretation appropriate?

Partly

Are all the source data underlying the results available to ensure full reproducibility?

Yes

Is the study design appropriate and is the work technically sound?

Yes

Are the conclusions drawn adequately supported by the results?

Partly

Are sufficient details of methods and analysis provided to allow replication by others?

Yes

Reviewer Expertise:

Endocrinology and PCOS

I confirm that I have read this submission and believe that I have an appropriate level of expertise to confirm that it is of an acceptable scientific standard, however I have significant reservations, as outlined above.

# Reviewer report: Reviewer response for version 5

**DOI:** 10.5256/f1000research.206706.r514927

**Published:** 2026-09-22

**Authors:** Erna Yovi Kurniawati

**Affiliations:** 1 Alma Ata University, Yogyakarta, Indonesia

Overall, the manuscript is well structured, with clear objectives, adequate methods and analysis, and findings that are relevant to the study aims. The results are clearly presented and appropriately supported by the analysis. As a minor enhancement, the authors are encouraged to further emphasize the **clinical implications** of the findings, particularly how the results may be translated into clinical practice, healthcare services, or clinical decision-making. Clarifying the potential practical application and contribution to improving the quality of care would further strengthen the manuscript's scientific and clinical relevance.

Is the work clearly and accurately presented and does it cite the current literature?

Yes

If applicable, is the statistical analysis and its interpretation appropriate?

Yes

Are all the source data underlying the results available to ensure full reproducibility?

Yes

Is the study design appropriate and is the work technically sound?

Yes

Are the conclusions drawn adequately supported by the results?

Yes

Are sufficient details of methods and analysis provided to allow replication by others?

Yes

Reviewer Expertise:

health reproductive, PCOS, Infertility, Endocrine Metabolic

I confirm that I have read this submission and believe that I have an appropriate level of expertise to confirm that it is of an acceptable scientific standard.

# Reviewer report: Reviewer response for version 4

**DOI:** 10.5256/f1000research.202995.r498042

**Published:** 2026-07-10

**Authors:** Mohd Ashraf Ganie, Nusrat Jahan

**Affiliations:** 1 Department of Endocrinology, Sheri Kashmir Institute of Medical Sciences, Srinagar, Jammu and Kashmir, India; 2 Sheri Kashmir Institute of Medical Sciences, Srinagar, Jammu and Kashmir, India

1. The diagnosis relied on clinical, laboratory, and ultrasound findings; however, standardized cut-off values for clinical hyperandrogenism (modified Ferriman–Gallwey score) and biochemical hyperandrogenism (serum testosterone) were not specified, which may limit the reproducibility and consistency of the diagnostic criteria.
2. Anti-Müllerian hormone (AMH) was mentioned as part of the diagnostic evaluation, but its values and diagnostic contribution were not reported or analyzed.
3. Some participants were receiving metformin, oral contraceptives, or vitamin D during sample collection, which may have influenced hormonal and immunological measurements despite meeting the diagnostic criteria for PCOS.
4. Other conditions that can mimic PCOS, such as non-classic congenital adrenal hyperplasia and Cushing syndrome, were not explicitly reported as being excluded, which may have introduced diagnostic heterogeneity.

Is the work clearly and accurately presented and does it cite the current literature?

Partly

If applicable, is the statistical analysis and its interpretation appropriate?

Yes

Are all the source data underlying the results available to ensure full reproducibility?

Yes

Is the study design appropriate and is the work technically sound?

Yes

Are the conclusions drawn adequately supported by the results?

Yes

Are sufficient details of methods and analysis provided to allow replication by others?

Partly

Reviewer Expertise:

Endocrine disorders especially PCOS

We confirm that we have read this submission and believe that we have an appropriate level of expertise to state that we do not consider it to be of an acceptable scientific standard, for reasons outlined above.

# Response

**Published:** 2026-07-12

**Authors:** Sura Alsaffar

**Affiliations:** Biology, University of Baghdad Department of Biology, Baghdad, Baghdad Governorate, Iraq

Dear reviewer, 

 ​​​​​​ Thanks for your time and your efforts in reviewing our paper, I'm pleased to answer your comments as the following:

  In regard to comments 1,2: The diagnosis depends on clinical investigation relied upon Rotterdam criteria, laboratory testing included (FSH, LH, Estrogen, Prolactin, Testosterone and AMH hormones) these hormones used for diagnosis as routine work but not mentioned in this paper.

 Modified Ferriman gallwey score wasn’t used by consultants.

 in regarding to comment 3

 Just 4 PCOS patients took the mentioned therapy, and the number of those patients wasn’t enough to put them in separate group, and I mentioned for scientific honesty 

 comment No 4 

 yes, true I didn’t mention in exclusion criteria, the diagnosis depends on hormones, symptoms and relied on ultrasound 

 I can add it for exclusion criteria for future studies as you recommended 

 The author 

 Dr Sura F Alsaffar 

 University of Baghdad, College of Science Department of Biology

# Response

**Published:** 2026-07-18

**Authors:** Hiba Khaleel

**Affiliations:** Biology, University of Baghdad Al-Jaderyia Campus College of Science, Baghdad, Baghdad Governorate, Iraq

Dear reviewer,

 Thank you for the feedback. All requested revisions have been successfully addressed and incorporated into the manuscript as detailed below:

 Response 1:

 We have explicitly specified the utilized BMI cut-off values (BMI < 25 for normal weight, 25-30 for overweight, and > 30 for obesity).

 Response 2:

 AMH was not the primary focus of this study.

 Response 3:

 We have clarified that only 4 participants were taking these medications, which did not affect the overall study outcomes.

 Response 4:

 We have clarified that only PCOS patients were included in this study.

# Reviewer report: Reviewer response for version 4

**DOI:** 10.5256/f1000research.202995.r498044

**Published:** 2026-07-08

**Authors:** Shahed Morshed

**Affiliations:** 1 Bangladesh Medical University,, Dhaka,, Bangladesh

The authors did not address the concerns.

**Abstract:**

 1. This is a case-control study. However, the authors tried to identify a causal association in the objective

 2. The objective was simply a comparison of the antibody levels between PCOS and control. The authors mentioned the role of antibodies in the occurrence of PCOS.

 3. The results section unnecessarily described the findings with LH & FSH. The correlation and association with BMI and LH-Ab and FSH-Ab were not described.

 4. The conclusion section should be mentioned according to the objectives. The authors unnecessarily described LH & FSH.

**Introduction:**

 1. Objective: Wrong hypothesis as previously described

**Methods:**

 1. Why is the sample size of the control less than the case? How did you match them?

 2. Wrong sampling technique

 3. How did you adjust the current treatment in your analysis?

 4. The sample size equation was not provided; it seems the wrong formula was used, and the minimum sample size was not mentioned.

 5. Diagnostic criteria: Mentioned in the abstract but not here. The operational definitions of each component were not described. LH/FSH ratio is not currently a diagnostic criterion in the Rotterdam criteria.

 6. Mention BMI cut-off to define overweight/obesity

 7. Gonadotropins instead of infertility hormones are more appropriate

 8. What is your CV of anti-LH and anti-FSH?

 9. Please correct: the chi-square test was used to compare the frequency between... 

 10. The power analysis should be done using your current sample size

 11. Mention the correlation analysis 

 12. How did you assume the normality of your numerical variables?

**Results:**

 1. The interpretation of the tables should be described, not the direct findings

 2. Table-1: The case and control groups are not matched by baseline characteristics, and the authors did not run an adjusted analysis.

 3.  Table 2:

 i. Repeated description of LH and LH-Ab.

 ii. anti-FSH: Non-significant increase in both groups; not clear how both groups?

 iii. No correlation was shown in Table -2, but described as "a negative correlation with LH levels"

 4. Table -3: Unnecessary

 5. Table 4: No statistical analysis was done. The sample size for subgroup analysis might be suboptimal.

 Discussion:

 1. The objective-oriented findings were not mentioned

 2. The discussion should be focused on the objectives, not LH and FSH

**Conclusion:**

 1. Not objective-based, not even according to the study findings.

 Due to several methodological inconsistencies, the article is not suitable for indexing.

Is the work clearly and accurately presented and does it cite the current literature?

Partly

If applicable, is the statistical analysis and its interpretation appropriate?

No

Are all the source data underlying the results available to ensure full reproducibility?

Partly

Is the study design appropriate and is the work technically sound?

No

Are the conclusions drawn adequately supported by the results?

Partly

Are sufficient details of methods and analysis provided to allow replication by others?

No

Reviewer Expertise:

PCOS, Diabetes, Obesity

I confirm that I have read this submission and believe that I have an appropriate level of expertise to state that I do not consider it to be of an acceptable scientific standard, for reasons outlined above.

# Response

**Published:** 2026-07-18

**Authors:** Hiba Khaleel

**Affiliations:** Biology, University of Baghdad Al-Jaderyia Campus College of Science, Baghdad, Baghdad Governorate, Iraq

Dear Reviewer,

 Response 1: Done. The wording in the abstract has been revised to focus purely on association rather than implying any causal relationship, fitting the case-control design.

 Response 2: Done. The objective in the abstract has been corrected to focus strictly on comparing antibody levels between the PCOS and control groups.

 Response 3: Done. General descriptions of LH and FSH have been removed from the abstracts' results. It now briefly details the correlation of BMI with LH-Ab and FSH-Ab.

 Response 4: Done. The conclusion of the abstract has been rewritten to align strictly with the study objectives. removing unnecessary general text about LH and FSH.

 Response 5: Done. The 2003 Rotterdam criteria have been explicitly defined in the methodology, and the LH/FSH ratio is now clarified as an exploratory.

 Response 6: Done. The WHO BMI cutoff classifications (< 25 and > 25 kg/m) have been added to the text.

 Response 7: Done. The term "infertility hormones" has been entirely replaced with "gonadotropins."

 Response 8: Done. The intra-assay and inter-assay coefficients of variation (CV) for both ELISA kits have been included in the methodology.

 Response 9: Done. The description of the chi-square test has been corrected in the statistical analysis section.

 Response 10, 12: Done. The Shapiro-Walk normality test and the post-hoc power analysis (>99%) details have been added to the statistical analysis section.

 Response 11: Done. Pearson correlation analysis has been explicitly mentioned and defined in the text.

 Results: Table 1, 2

 The descriptions have been revised to correct numerical overlaps, remove incorrect correlation terms, and properly reflect the adjusted age matching (p=0.660) and non-significant fertility values (p=0.830).

 Response to conclusion: The conclusion has been rewritten.

# Reviewer report: Reviewer response for version 3

**DOI:** 10.5256/f1000research.198200.r481530

**Published:** 2026-05-22

**Authors:** Shahed Morshed

**Affiliations:** 1 Bangladesh Medical University,, Dhaka,, Bangladesh

The research methodology is not replicable.

 1. The sampling technique seems a convenient sampling. The authors repeatedly mention random sampling. Needs explanation about the sampling frame and the type of random sampling.

 2. The authors did not provide a sample size calculation or the power of the study with the current sample size.

 3. The authors did not provide the diagnostic criteria used to define PCOS .

 4. Those taking medications may alter the antibody levels; they did not mention the number of patients. Any adjustment by regression was not attempted .

 5. Statistical analysis: t-test and chi-square tests are not used to compare percentages.

 6. Table 3 and 4: Statistical tests name- Absent

 7. The method of reporting p-values is not uniform.

Is the work clearly and accurately presented and does it cite the current literature?

Partly

If applicable, is the statistical analysis and its interpretation appropriate?

No

Are all the source data underlying the results available to ensure full reproducibility?

Partly

Is the study design appropriate and is the work technically sound?

No

Are the conclusions drawn adequately supported by the results?

Partly

Are sufficient details of methods and analysis provided to allow replication by others?

No

Reviewer Expertise:

PCOS, Diabetes, Obesity

I confirm that I have read this submission and believe that I have an appropriate level of expertise to state that I do not consider it to be of an acceptable scientific standard, for reasons outlined above.

# Response

**Published:** 2026-05-28

**Authors:** Hiba Khaleel

**Affiliations:** Biology, University of Baghdad Al-Jaderyia Campus College of Science, Baghdad, Baghdad Governorate, Iraq

Dear Reviewer,

 Thank you for the comment.

 1. The sampling technique has been corrected and clarified as convenience sampling throughout the manuscript.

 2. A post hoc power analysis was performed and confirmed that the sample size was adequate to detect significant differences between the study groups.

 3. The diagnostic criteria for PCOS based on Rotterdam criteria (2003) have been added to the methodology section.

 4. The use of medications has now been clarified in the manuscript. only 4 patients, the results not affected.

 5. The statistical analysis section has been revised to clearly specify the appropriate statistical tests used for each comparison.

 6. The names of the statistical tests used in tables 3 and 4 have now been added.

 7. The reporting format of P-values has been standardized throughout the manuscript.

# Reviewer report: Reviewer response for version 2

**DOI:** 10.5256/f1000research.196070.r467849

**Published:** 2026-03-25

**Authors:** Shahed Morshed

**Affiliations:** 1 Bangladesh Medical University,, Dhaka,, Bangladesh

Major correction:

 1. Sampling technique: 'Random' is not clear. Please explain in details

 2. Please provide the sample size calculation

 3. The diagnostic criteria and the definition of each component are not described. LH/FSH ratio is not currently used in the diagnosis of PCOS. Diagnosis of PCOS also required exclusion of similar endocrinopathies, not described.

 4. The study was conducted in an uncontrolled condition where some got different types of drugs. The number, dose, and amount are not described. How did these drugs affect the findings? They were not adjusted in the analysis.

 5. Please provide details about the statistical analysis. An independent-samples test is conducted to compare means, not percentages.

 6. Table 1: Describe the interpretations rather than simply describing the tables.

 7. In the footnotes of all tables: Provide the name of statistical tests.

 8. Table 4: No statistical analysis was shown

 9. Table 5: Two columns with r- could not understand

 10. Discussion: 'In line with this, the current study observed a negative correlation between LH and

 anti-LH Abs, supporting the proposed relationship': No correlation between LH and anti-LH Ab is provided

 Minor

 Abstract: 

 1. Replace 'about' with 'A total of'

 2. Correct age range

Is the work clearly and accurately presented and does it cite the current literature?

Partly

If applicable, is the statistical analysis and its interpretation appropriate?

No

Are all the source data underlying the results available to ensure full reproducibility?

Partly

Is the study design appropriate and is the work technically sound?

No

Are the conclusions drawn adequately supported by the results?

Partly

Are sufficient details of methods and analysis provided to allow replication by others?

No

Reviewer Expertise:

PCOS, Diabetes, Obesity

I confirm that I have read this submission and believe that I have an appropriate level of expertise to state that I do not consider it to be of an acceptable scientific standard, for reasons outlined above.

# Response

**Published:** 2026-03-28

**Authors:** Hiba Khaleel

**Affiliations:** Biology, University of Baghdad Al-Jaderyia Campus College of Science, Baghdad, Baghdad Governorate, Iraq

Dear reviewer,

 Thank you for your valuable comments and suggestions. We have carefully revised the manuscript in accordance with your feedback.

 Comment 1 response: We have clarified the sampling method in the method section.

 Comment 2 response: The sample size calculation has now been added to the methods section, including the formula and parameters used.

 Comment 3 response: The diagnostic criteria have been revised according to standard guidelines, and relevant details have been added. We also clarified the exclusion of other endocrine disorders.

 Comment 4 response: We have clarified that some participants were receiving medication (e.g., metformin and oral contraceptives) recently during sample collection, but they still meet PCOS criteria as diagnosed by a specialist.

 Comment 5 response: The statistical methods have been revised and clearly stated. Appropriate tests were used based on data type, and all tables now include the names of statistical tests and p-values.

 Comment 6 response: Interpretation of the findings has been added to the results section.

 Comment 7 response: All tables have been updated to include the appropriate statistical test in the footnotes.

 Comment 8 response: Statistical analysis, including p-values, has been added to table 4.

 Comment 9 response: Table 5 has been revised to clearly indicate the type of correlation (Pearson), and column descriptions have been added.

 Comment 10 response: The discussion section has been revised.

 Minor comments (Abstract): The abstract has been revised to include a total and an age range.

 We appreciate your insightful comments, which have significantly improved the quality of our manuscript.

 sincerely,

 Hiba Muhammed Khaleel.

 University of Baghdad/College of Science.

# Reviewer report: Reviewer response for version 2

**DOI:** 10.5256/f1000research.196070.r465133

**Published:** 2026-03-12

**Authors:** Mohd Ashraf Ganie, Nusrat Jahan

**Affiliations:** 1 Department of Endocrinology, Sheri Kashmir Institute of Medical Sciences, Srinagar, Jammu and Kashmir, India; 2 Sheri Kashmir Institute of Medical Sciences, Srinagar, Jammu and Kashmir, India

Despite providing valuable insights into the Serological Evaluation of Anti- FSH Antibody and Anti- LH antibody In Iraqi women with polycystic ovarian syndrome, this study has several limitations that should be considered when interpreting the findings.

1. The study included only 65 patients with PCOS and 56 controls, which may limit the statistical power and generalizability of the findings. A larger sample size would allow more robust analysis and improve the reliability of associations between anti-gonadotropin antibodies and PCOS.
2. Participants were recruited from Medical City Hospital and a few private infertility clinics in Baghdad, which may introduce selection bias. Therefore, the results may not represent the broader population of women with PCOS from different geographic or ethnic backgrounds.
3. Some participants were receiving treatments such as metformin, vitamin D, and oral contraceptives at the time of sample collection. These medications can influence LH, FSH, and the LH/FSH ratio, potentially affecting the interpretation of hormonal and immunological results.
4. The study evaluated only anti-FSH and anti-LH antibodies. Other immune markers and inflammatory cytokines that may contribute to autoimmune mechanisms in PCOS were not assessed, limiting a comprehensive understanding of the immune involvement in the disorder.
5. Because the study used a cross-sectional design, it cannot establish causal relationships between anti-gonadotropin antibodies and the development or progression of PCOS. Longitudinal studies are needed to determine whether these antibodies contribute directly to disease pathogenesis.
6. Key metabolic parameters commonly associated with PCOS, such as insulin resistance, lipid profile, and glucose tolerance, were not evaluated. Including these parameters could provide a more comprehensive picture of endocrine-metabolic interactions.
7. Although BMI was analyzed, the study only categorized participants into BMI >25 and <25, which may not fully capture variations in adiposity or metabolic risk. More detailed anthropometric or metabolic assessments could improve the analysis.

Is the work clearly and accurately presented and does it cite the current literature?

Partly

If applicable, is the statistical analysis and its interpretation appropriate?

Yes

Are all the source data underlying the results available to ensure full reproducibility?

Yes

Is the study design appropriate and is the work technically sound?

Yes

Are the conclusions drawn adequately supported by the results?

Yes

Are sufficient details of methods and analysis provided to allow replication by others?

Partly

Reviewer Expertise:

Endocrine Disorders

We confirm that we have read this submission and believe that we have an appropriate level of expertise to confirm that it is of an acceptable scientific standard, however we have significant reservations, as outlined above.

# Reviewer report: Reviewer response for version 1

**DOI:** 10.5256/f1000research.189997.r441802

**Published:** 2025-12-31

**Authors:** Taieb Ach

**Affiliations:** 1 Laboratory of Exercise Physiology and Pathophysiology, Sousse, Tunisia

The authors reported the use of antiFSH and antiLH in PCOS patients , and their influence on the clinical, hormonal and diagnosis aspects.

 The research is original with novel results. 

 This is a well written manuscript , with updated references.

 Here are my comments to improve it before acceptance :

 - Introduction is too long with a lot of references. The authors should state mainly the originality of the research.

 - AMH sampling is missing the diagnosis criteria.

 - The authors could update the number of follicles diagnosis in MENA region with a recent north african cutoff :( ref 1 )

 - Add more details on PCOS phenotypes , and diagnosis criteria

 - Patients sampling is missing

 - Why in the results : androgen sampling is missing. 

 - Is it necessary to have three numbers after decimals in each sampling ?

 - Add abbreviations under tables : NS etc ..

 - The results are original , however ; clinical implications are not welle explained.

 Add more details on cost, clinical applications.

 - Strength and limitations must be enhanced.

Is the work clearly and accurately presented and does it cite the current literature?

Partly

If applicable, is the statistical analysis and its interpretation appropriate?

Yes

Are all the source data underlying the results available to ensure full reproducibility?

Yes

Is the study design appropriate and is the work technically sound?

Yes

Are the conclusions drawn adequately supported by the results?

Yes

Are sufficient details of methods and analysis provided to allow replication by others?

Partly

Reviewer Expertise:

Reproductive Endocrinology

I confirm that I have read this submission and believe that I have an appropriate level of expertise to confirm that it is of an acceptable scientific standard, however I have significant reservations, as outlined above.

## References

[1] : Validation of the follicular and ovarian thresholds by an 18-MHz ultrasound imaging in polycystic ovary syndrome: a pilot cutoff for North African patients. *Therapeutic Advances in Reproductive Health* .2024;18: 10.1177/26334941241270372 10.1177/26334941241270372 PMC1133717639170801

# Response

**Published:** 2026-02-01

**Authors:** Hiba Khaleel

**Affiliations:** Biology, University of Baghdad Al-Jaderyia Campus College of Science, Baghdad, Baghdad Governorate, Iraq

Comment 1:

 The response: The introduction has been shortened, and the originality regarding the role of anti-FSH antibody and anti-LH antibody in PCOS diagnosis and clinical implications has been preserved.

 Comment 2: 

 The response: The diagnostic criteria have been clarified under the Materials and Methods section. The role of AMH measurement in relation to these criteria has been clarified.

 Comment 3:

 The response: The study was conducted exclusively on Iraqi women.

 Comment 4:

 The response: PCOS phenotypes were not specified in this study; it is beyond the scope of the study.

 Comment 5:

 The response: The patients' sampling has been included.

 Comment 6:

 The response: Androgen levels were not assessed in this study, as the focus was on other diagnostic criteria.

 Comment 7:

 The response: Yes, using 3 decimal places ensures higher precision and consistency in reporting the sampling data.

 Comment 8:

 The response: All abbreviations used in the table, e.g., (NS: Not Significant), have been clearly defined in the table footnotes. 

 Comment 9: 

 The response: The discussion section has been expanded to better explain the clinical implications of the findings. Additional details regarding potential cost-effectiveness and practical clinical applications of using anti-FSH and anti-LH antibodies in PCOS diagnosis have been included.
